# Microbiome-Based Products: Therapeutic Potential for Inflammatory Skin Diseases

**DOI:** 10.3390/ijms26146745

**Published:** 2025-07-14

**Authors:** Anamarija Rušanac, Zara Škibola, Mario Matijašić, Hana Čipčić Paljetak, Mihaela Perić

**Affiliations:** 1 Department of Intercellular Communication, Center for Translational and Clinical Research, School of Medicine, University of Zagreb, Šalata 2, 10000 Zagreb, Croatia; anamarija.rusanac@mef.hr (A.R.); zara.skibola@mef.hr (Z.Š.); hana.paljetak@mef.hr (H.Č.P.); mihaela.peric@mef.hr (M.P.); 2 BIMIS—Biomedical Research Center Šalata, School of Medicine, University of Zagreb, 10000 Zagreb, Croatia

**Keywords:** dysbiosis, human microbiota, inflammatory skin disease, microbiome-based products

## Abstract

Maintaining a balanced skin microbiota is essential for skin health, whereas disruptions in skin microbiota composition, known as dysbiosis, can contribute to the onset and progression of various skin disorders. Microbiota dysbiosis has been associated with several inflammatory skin conditions, including atopic dermatitis, seborrheic dermatitis, acne, psoriasis, and rosacea. Recent advances in high-throughput sequencing and metagenomic analyses have provided a deeper understanding of the skin microbial communities in both health and disease. These discoveries are now being translated into novel therapeutic approaches aimed at restoring microbial balance and promoting skin health through microbiome-based interventions. Unlike conventional therapies that often disrupt the microbiota and lead to side effects or resistance, microbiome-based products offer a more targeted strategy for preventing and managing inflammatory skin diseases. These products, which include probiotics, prebiotics, postbiotics, and live biotherapeutic agents, are designed to modulate the skin ecosystem by enhancing beneficial microbial populations, suppressing pathogenic strains, and enhancing immune tolerance. As a result, they represent a promising class of products with the potential to prevent, manage, and even reverse inflammatory skin conditions. However, realizing the full therapeutic potential of microbiome-based strategies in dermatology will require continued research, robust clinical validation, and clear regulatory frameworks.

## 1. Introduction

The human body is inhabited by trillions of microorganisms, collectively known as the microbiota, residing in highly symbiotic and interdependent relationships both mutually and with their host. Microbiota composition varies across different regions of the body and is shaped by factors such as the environmental niche, the host’s immune status, and interactions between microbial species [1,2]. Over the past decade, there has been a significant expansion in microbiota research, leading to a more comprehensive understanding of its structural and functional dynamics. This progress recognized the importance of the microbiota in human health and opened opportunities for the development and application of microbiome-based products with therapeutic potential.

The skin serves as the body’s primary physical barrier against the external environment, protecting against pathogens, dehydration, UV light, and physical damage. It also hosts diverse microorganisms—bacteria, fungi, viruses, and archaea—that form communities supporting the immune system and defending against harmful microbes [3]. Maintaining a balanced skin microbiota is crucial for skin health while disruptions in skin microbiota composition, referred to as dysbiosis, can contribute to the development and progression of various diseases. Microbiota dysbiosis has been associated with several inflammatory skin conditions, including atopic dermatitis (AD), seborrheic dermatitis (SD), acne, psoriasis, and rosacea [2,4,5,6,7].

Members of the microbiota are being explored for diverse applications, including the treatment of dermatological and gastrointestinal conditions, as well as in improving skincare [2,8]. Although these approaches show significant promise, extensive human trials are necessary to evaluate potential risks, confirm their efficacy, and fully understand their direct and indirect effects. This review provides a summary of the key information about the human skin microbiota, its connection to inflammatory skin diseases, and its potential use as therapeutics for these conditions. To ensure clarity and consistency, the key terms used throughout this manuscript are defined in Table 1.

## 2. The Human Skin Microbiota

The skin, comprising approximately 15% of the adult body weight, functions as a complex barrier organ with physical, chemical, and immunological roles, while also supporting a site-specific microbiota through the provision of nutrients such as amino acids, fatty acids, and lactic acid [3,16]. It is well established that different microbial species, along with their structural components and metabolites play a crucial role in protecting against pathogenic or harmful microorganisms and maintaining skin homeostasis, modulating skin immunity, stimulating and activating various immune responses, metabolizing natural products, and producing antimicrobial peptides (AMPs) [2,17,18]. The initial skin microbiota colonization begins at birth and is strongly influenced by the mode of delivery and the maternal microbiota to which neonates are exposed during labour [6,16]. Throughout life, the microbiota composition is affected by various intrinsic factors, such as the skin site, age, ethnicity, gender, and intra- and interpersonal variability, as well as extrinsic factors, including the mode of delivery, lifestyle, living and working environment, diet, antibiotic exposure, hygiene practices, skin disinfection, cosmetic use, clothing textiles, climate, seasonality, and geographical location [2,4,19].

The normal human skin microbiota is predominantly composed of the bacterial phyla *Actinobacteria* (52%), *Firmicutes* (24%), *Proteobacteria* (17%), and *Bacteroidetes* (6%) but new taxa are being identified as methodological approaches advance [20]. The dominant genera include *Corynebacterium*, *Cutibacterium* (previously known as *Propionibacterium*), and *Staphylococcus* genera that comprise more than 60% of the bacterial skin population [16,21,22,23]. The skin also hosts various fungal species, such as *Malassezia* spp., *Cryptococcus* spp., *Rhodotorula* spp., *Aspergillus* spp., and *Epicoccum* spp., with *Malassezia* spp. being the most prevalent, comprising approximately 80% of the total fungal microbiota [4,5,6,16,24,25]. Several studies also indicate the presence of *Candida* species in the normal skin mycobiome [26,27]. However, fungi represent the least abundant microorganisms on the skin [28]. Viruses within the skin microbiota have been less explored than cellular microorganisms but a recent study identified a large set of viral metagenome-assembled genomes (vMAGs) on several body sites [20]. Among them, cutaneous human papillomaviruses are commonly found but in lower abundance compared to the bacteriophages which are linked to the modulation of bacterial populations and influence skin microbiota homeostasis [29,30]. *Cutibacterium* and *Staphylococcus* phages are the most abundant skin phages followed by *Streptococcus* and *Corynebacterium* phages [28]. Additionally, there are small mites from the *Demodicidae* family that naturally inhabit sebaceous areas of the human skin, such as the face and hair follicles. Specifically, two species have been isolated from human samples: *Demodex folliculorum* and *Demodex brevis* [29,31].

**Figure 1:**
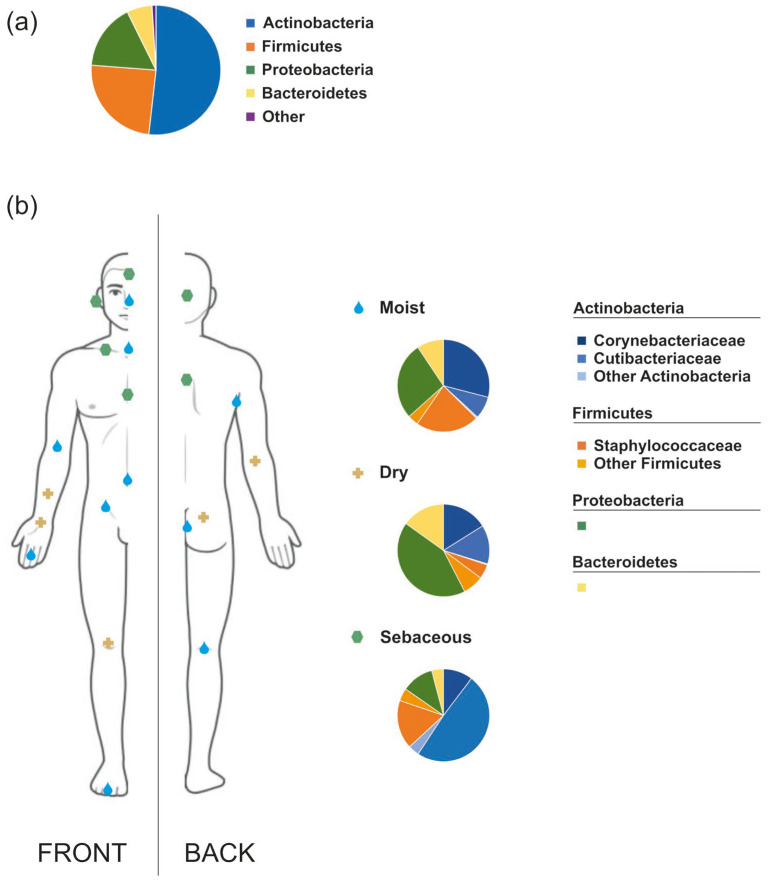
(**a**) General overall composition of normal human skin microbiota; (**b**) Difference in composition of microbiota across different parts of human skin: moist, dry and sebaceous. Figure based on data from Grice et al. [32].

The composition of the human skin microbiota is relatively stable, though it varies according to the physiological traits of different body regions: sebaceous (e.g., face, chest, and back), moist (e.g., elbows, knees, and genitalia), and dry (e.g., palms) (Figure 1) [6,21,24,32]. Sebaceous (oily) areas show the lowest microbial diversity and are primarily inhabited by lipophilic microorganisms, such as *Cutibacterium*, *Staphylococci*, and *Corynebacterium* [21,24,33]. Moist skin areas, providing a thermally stable and warm environment, are dominated by *Corynebacterium* and *Staphylococcus* species, which are well-adapted to survive in these conditions [3,21]. Dry skin areas, by contrast, are among the most diverse sites on the human body in terms of microbial composition. Despite this diversity, dry skin is predominantly colonized by bacteria from the genera *Corynebacterium*, *Flavobacteriales*, and *Proteobacteria* [34].

## 3. Skin Microbiota Dysbiosis and Inflammatory Skin Diseases

Although the exact mechanisms underlying the maintenance and regulation of skin homeostasis remain unclear, it is well-established that the balance among the members of the skin microbiota plays a critical role in protecting against skin diseases [2]. Several chronic inflammatory skin diseases (ISDs), such as atopic dermatitis, seborrheic dermatitis, acne, psoriasis, and rosacea, are associated with skin microbiota dysbiosis [2,3,4,5,6,7,35]. These conditions are most commonly related to increased levels of *Staphylococcus aureus* and a decrease in *Cutibacterium* species. However, each disease has its own specific characteristics and microbial shifts that define its unique dysbiosis profile, which will be discussed in detail below. Table 2 summarizes the current knowledge of the main ISDs and the alterations in the human skin microbiota, presented as an increase or decrease in bacterial genera, species, or species ratios.

Beyond the skin microbiota dysbiosis paradigm, the skin-gut axis hypothesis proposes that the immune system, metabolic-hormonal pathways, and nervous system all play a role in linking gastrointestinal health and skin health [56]. The gut microbiota, as the primary modulator of the gut-skin axis, affects immunity, and a dysbiosis of the gut disrupts the immune system’s equilibrium [57]. According to the gut-skin axis hypothesis, inflammatory skin conditions are caused by a complex interaction between the immune system, lifestyle, and genetics that is constantly synchronized with the skin neuro–immuno–endocrine system [58,59]. The gut and cutaneous microbiota are important in these connections because the immune system and microbes are constantly interacting in the colon and on the skin [57]. Many skin disorders coexist with non-cutaneous conditions like gastrointestinal diseases, further supporting the interdependence of the skin and the gut.

### 3.1. Atopic Dermatitis

Atopic dermatitis, also known as eczema, is the most common cause of skin illness worldwide with a prevalence of 2.6%, affecting over 200 million people [60]. The hallmark of the disease is dry, red and itchy skin which typically first appears in childhood but can occasionally reappear in adulthood. The pathophysiology of AD remains an open debate. According to the external hypothesis, a basic barrier malfunction (such as a loss-of-function mutation in barrier genes like FLG, that codes for filaggrin) results in a defective epidermal barrier and increased allergen penetration, which in turn triggers the skin immune system and causes atopic inflammation [61]. In contrast, the inside-out hypothesis suggests that skin inflammation develops due to a primary immune defect, which in turn causes a secondary barrier malfunction, microbial dysbiosis and allergen penetration [62].

The skin of AD patients exhibits significant alterations in microbial communities compared to that of healthy individuals, marked by pronounced microbial imbalance and decreased diversity. This is manifested by the significant reduction in *Cutibacterium*, *Streptococcus*, *Acinetobacter*, *Corynebacterium*, and *Prevotella*, together with a marked overrepresentation of *Staphylococcus*, especially *S. aureus* [37]. Indeed, *S. aureus* is the primary bacterial species linked to AD, with its dominance especially pronounced during severe disease flares. In these instances, microbial diversity significantly decreases, often resulting in the dominance of a single *S. aureus* strain [6]. The key event in *S. aureus* skin colonization is its adhesion to corneocytes by binding to corneodesmosin, a glycoprotein adhesion molecule displayed on the surface of corneocytes. Due to the low levels of skin humectants and moisturizing factors in AD, corneocytes excessively display corneodesmosin and facilitate the binding of *S. aureus* [63]. *S. aureus* expresses numerous secreted and wall-anchored virulence factors, e.g., superantigens (toxins), enzymes, and other proteins, thereby compromising the skin barrier and allowing allergen presentation to epidermal dendritic cells, which intensifies AD symptoms [64]. The severity of the condition also correlates with a decrease in coagulase-negative species and a decrease in microbiota diversity [30,65]. Patients with AD tend to have reduced levels of protective commensal skin bacteria, specifically coagulase-negative *Staphylococcus* species like *Staphylococcus epidermidis* and *Staphylococcus hominis*, which shield healthy skin against harmful pathogens by secreting lantibiotics or other antimicrobial peptides (AMPs). Reintroducing these antimicrobial coagulase-negative strains to AD patients decreased *S. aureus* colonization, indicating that skin microbiota dysbiosis plays a role in the disease pathology [66].

However, it is still insufficiently clear how staphylococci contribute to the development of AD. To determine if elevated staphylococci levels precede clinical symptoms, one study monitored newborns by swabbing four distinct skin sites at three timepoints. The findings have shown that infants that developed AD by 12 months of age had significantly lower levels of commensal staphylococci present at 2 months compared to healthy controls, indicating that early exposure to commensal staphylococci may support the development of a healthy immune system and provide protection against the onset of disease later in life [67]. Similarly, another study found that infants who later developed AD had elevated levels of *S. aureus* on their skin at 3 months of age. Compared to unaffected, age-matched infants, *S. aureus* was more prevalent both at the time of AD onset and two months prior [68]. In contrast to *S. aureus*, *S. epidermidis* has been considered a skin commensal organism, essential in combating pathogens. Recent results, however, propose that *S. epidermidis* can contribute to the inflammatory reaction in AD, with its cysteine protease, EcpA, compromising the skin barrier. On the other hand, it was demonstrated that *S. hominis*, another commensal skin bacterium species, inhibits the *S. epidermidis* production of EcpA, highlighting the importance of interspecies interactions and the necessity of individualized and multitargeted therapeutic approaches in AD [69].

Compared to the extensive research on skin bacteria, studies examining the diversity of skin fungi in AD remain limited. Similarly to healthy individuals, *Malassezia* species, particularly *Malassezia globosa* and *Malassezia restricta*, are predominant in patients with AD [27]. In patients with mild to moderate AD, *M. restricta* tends to be more abundant than *M. globosa*, whereas in severe cases, both species are present in roughly equal proportions [27,70]. It has been demonstrated that *Malassezia* can penetrate the impaired epithelial barrier in AD, triggering immune cell activation and skin inflammation. Moreover, allergens produced by *Malassezia* can elicit a specific IgE-mediated immune response, further contributing to the pathogenesis of the disease [71].

A recent study by Wielscher et al. demonstrated that healthy and AD skin harbour distinct phageomes suggesting a causative relationship between shifts in viral and bacterial communities and the development of skin pathology. Despite notable inter-individual variability in phage composition, the study identified increased *S.aureus*-specific phages in patients with AD, indicating that these could provide a growth advantage for *S. aureus*. On the other hand, the abundance of certain phages decreased with increasing disease severity implying a possible skin protective role [30].

### 3.2. Seborrheic Dermatitis

Seborrheic dermatitis is characterized by itchy, red patches and greasy scales, typically affecting areas abundant in sebaceous glands—the scalp, face and upper chest. SD affects up to 5% of the general population, with a higher prevalence in immunocompromised individuals and those with neurological conditions [72]. Because of its multifactorial etiology it is very difficult to establish the primary origin of the disease, with its most prominent pathology factors including immune responses, skin microbiota dysbiosis and increased sebocyte activity [73].

Recent advances in microbiota research revealed an imbalance of fungal and bacterial communities on the skin of SD affected individuals [74]. It has been proposed that *Malassezia* spp. play an important role in the initiation of SD and are crucial contributors to the disease severity [74,75]. Although a part of the normal skin microbiota, predominantly residing on sebaceous areas, *Malassezia* can produce lipases and phospholipases that decompose sebum and release unsaturated fatty acids and lipid peroxides causing inflammation [76]. The activation of keratinocyte pattern recognition receptors triggers the NF-κB and MAPK pathways, leading to the release of cytokines from immune cells and the promotion of inflammation [77]. This leads to keratinocyte proliferation, stratum corneum thickening, and barrier disruption. A compromised barrier facilitates *Malassezia* invasion, resulting in transepidermal water loss, skin peeling, and other SD symptoms caused by ceramide depletion [78]. It has been demonstrated that *Malassezia* spp. host dsRNA mycoviruses, which can enhance fungal gene expression, and the presence of these mycoviruses is associated with a greater disease severity. Also, higher levels of *Malassezia* are linked to increased itching scores and greater disease severity, highlighting its role in disease manifestation [79].

In addition, *Malassezia*-derived unsaturated fatty acids increase skin pH, promoting *S. aureus* growth and its adhesion to keratinocytes. Indeed, *S. aureus* has been identified as the most prevalent bacterium on SD-affected skin, occurring significantly more often than in healthy controls [79,80]. Microbiota studies have shown that *Cutibacterium* is more abundant in non-lesional skin, whereas *Staphylococcus* and *Streptococcus* dominate lesional areas [81]. Similarly, Tanaka et al. found increased *Cutibacterium* at non-lesional sites and elevated *Acinetobacter*, *Staphylococcus*, and *Streptococcus* at lesional ones based on qPCR [41]. These sebum-degrading bacteria, together with *Malassezia*, may contribute to SD pathogenesis. Elevated *Staphylococcus* levels correlate with increased transepidermal water loss and higher skin pH, indicating barrier disruption, while *Cutibacterium* is associated with better hydration and barrier integrity [82]. Supporting this, a recent study reported scalp microbiota dysbiosis in SD, with a reduction in *Corynebacterium* and a predominance of *Pseudomonas*, *Staphylococcus*, and *Micrococcus* sp. [40].

### 3.3. Acne Vulgaris

One of the most prevalent dermatological disorders in the world, acne vulgaris affects the pilosebaceous unit, which includes the hair follicle, hair shaft, and sebaceous gland [83]. *Cutibacterium acnes*, the predominant organism in sebaceous skin areas, is linked to acne vulgaris [84]. Increased sebum production and changes in the fatty acid composition of sebum, hormonal microenvironment dysregulation, interaction with neuropeptides, follicular hyperkeratinization, inflammation induction, and innate and adaptive immune system dysfunction are some of the major mechanisms that contribute to the development of acne [85]. Despite similar relative abundances of *C. acnes* in the two cohorts (patients and healthy controls), metagenomic research revealed that population structures at the strain level differed dramatically. While certain strains were abundant in healthy skin, others were strongly linked to acne. Therefore, it appears that a loss of diversity and the predominance of phylotype IA1 and, to a lesser extent, phylotype IA2 (among six phylogenetic groups IA1, IA2, IB, IC, II, and III) contribute more to acne development than the relative abundance of *C. acnes* on the skin [43,85]. One study found that individuals with and without acne have different *C. acnes* gene expression profiles and proposed a mechanism of vitamin B12-mediated acne pathogenesis. Elevated levels of vitamin B12 in the host causes the inhibition of vitamin B12 production and elevated 2-oxoglutarate and L-glutamate levels in *C. acnes*. Due to the increased flux of L-glutamate to the porphyrin biosynthetic pathway *C. acnes* produces an excess of porphyrins. This triggers an inflammatory response in the host, contributing to the development of acne [86]. Dreno et al. showed that the severity of acne is correlated with an increase in *Staphylococcus* abundance [42]. However, commensal staphylococci have a crucial role in maintaining the equilibrium of *C. acnes* in healthy human skin. It has been demonstrated that microbial interference contributes to the homeostasis of healthy skin, and certain strains of *Staphylococci* may have anti-*C. acnes* properties [87]. *S. epidermidis* can prevent the growth of *C. acnes* [42] and reduce the skin irritation it causes [88]. *S. epidermidis* enhances the fermentation of naturally occurring skin glycerol by producing succinic acid, a byproduct of fatty acid fermentation, thus suppressing the *C. acnes* proliferation [89]. Toll-like receptor (TLR)-2 production is inhibited by lipoteichoic acid, which mediates the anti-inflammatory actions of *S. epidermidis*. In this way, *S. epidermidis* can inhibit the *C. acnes*-induced production of tumour necrosis factor (TNF)-alpha and IL-6 in keratinocytes [88].

Additionally, differences in *C. acnes* phage abundance have been observed between healthy individuals and acne patients [45] associating phage deficiency with acne development. Furthermore, a direct relationship between *C. acnes* phage abundance and age has been reported, suggesting that phage abundance may play a role in the reduced prevalence of acne vulgaris among ageing individuals.

### 3.4. Psoriasis

Psoriasis is triggered and sustained by Th17 cells and the production of their associated proinflammatory mediators. The hallmarks of the disease are flaky skin patches and scales due to the excessive keratinocyte proliferation and redness from dilated dermal blood vessels and immune cell infiltration [90]. While the precise etiology and pathology of the disease are still unknown, recent studies revealed an association between skin microbiota dysbiosis and psoriasis.

A significant reduction in the alpha- and beta-diversity of the skin microbiota was reported in patients with psoriasis, with lower abundance of *Cutibacterium*, *Burkholderia*, and *Lactobacilli*, along with increased levels of *Corynebacterium kroppenstedii*, *Corynebacterium simulans*, *Neisseria* spp., and *Finegoldia* spp. compared to healthy individuals [46,91]. The microbiota dysbiosis in psoriasis is characterized by elevated levels of the *Staphylococcus* genus increased in both lesional and non-lesional skin of patients compared to healthy controls [47,92]. *S. aureus* colonizes psoriatic skin lesions in almost 60% of patients, compared to 5% to 30% in healthy controls, with the generation of the staphylococcal enterotoxin highly correlated with the disease severity [93]. A study also reported that colonization by *S. aureus* sets off an inflammatory Th17 response sustaining keratinocyte proliferation, promoting the infiltration of immune cells and subsequently amplifying the inflammation process [47].

*Streptococcus* is the most common bacterial genus identified in psoriatic skin, with *Streptococcus pyogenes* infection of the pharynx recently recognized as a risk factor for psoriasis development [49]. *S. pyogenes* can produce streptococcal M protein and streptococcal peptidoglycan, functioning as superantigens that activate circulating cutaneous lymphocyte-associated antigen (CLA)-positive (+) memory T cells [94]. The activation of CLA+ T cells leads to the increased production of IL-17, the cytokine recognized as the key driver of psoriasis [95].

A variety of fungi in psoriatic skin have been found to trigger the disease by activating the innate immune system [96]. A meta-analysis by Pietrzak et al. reported higher levels of *Candida* spp. in psoriasis patients compared to controls [97]. In addition to *C. albicans*, members of the *Malassezia* genus were also found to aggravate psoriasis, even though no clear correlation was found between them and psoriatic lesions in general [48].

According to several studies, the dysbiosis linked to psoriasis may have its origins in the intestine. The findings that are most commonly reported are the increase in *Firmicutes* and *Actinobacteria* and the decrease in *Bacteroides* and *Akkermansia* spp. [75,98]. Bacterial translocation in the altered intestine causes inflammation and an abnormal microbiome, which in turn causes the inflammatory response to persist [99].

In a recent study, alterations in the phageome composition were correlated with psoriasis lesional skin [50] with the most abundant phage species showing decreased abundance in lesional areas compared to healthy skin from contralateral sites as well as family controls, indicating the state of dysbiosis of psoriatic skin and the potential effect of phages on bacterial diversity.

### 3.5. Rosacea

Rosacea is characterized by recurring facial erythema, pustules and papules, telangiectasia (visible blood vessels), and face flushing. Rosacea primarily affects the forehead, chin, nose and cheeks, with the disease alternating between flare-ups and remissions [100]. The current hypothesis suggests that the dysbiosis of the skin microbiota contributes to the disease pathophysiology by stimulating the innate immunity [101]. The dysbiosis of the skin microbiota in rosacea is primarily reflected in the increased load of *Demodex* mites, particularly *Demodex folliculorum* [51,54,102]. *Demodex* mites may have a role in the early inflammatory phase in rosacea by secreting bioactive compounds and influencing immunological responsiveness in sebocytes through the upregulation of TLR2 expression and elevated IL-8 [103]. Furthermore, the *Demodex* mite is an endosymbiont vector, and various bacterial species including *S. epidermidis*, *Chlamydophila pneumoniae* and several *Bacillus* species, including *Bacillus oleronius*, *Bacillus cereus*, *Bacillus pumilus* and *Bacillus simplex*, have been isolated from *D. folliculorum* [51].

The Gram-negative bacterium *B. oleronius* was initially identified in a *D. folliculorum* mite isolated from a rosacea patient’s face [104]. *B. oleronius* displayed antigens that considerably increased the peripheral blood mononuclear cell proliferation in rosacea patients compared to healthy controls, activating the production of proinflammatory cytokines and driving neutrophil recruitment [105]. Recently, a novel mechanism of rosacea pathogenesis was proposed, which includes microbial dysbiosis and a higher abundance of *B. oleronius,* inducing a proinflammatory response and triggering neoangiogenesis [106]. In addition, elevated temperature in the skin affected by rosacea can influence other bacterial communities and their metabolism. For instance, *S. epidermidis*, typically considered a commensal, can start behaving as a pathogen in altered skin conditions, generating virulence factors which are absent in individuals who are not rosacea-affected [107], suggesting that *S. epidermidis* can also play a role in the disease development [108].

According to a recent study, *C. kroppenstedtii* and *D. folliculorum* exist in a mutualistic relationship, and viable bacteria appear to be a requirement for the survival of *Demodex* mites [109]. Rainer et al. discovered that *C. kroppenstedtii* was one of the most prevalent bacteria and *Roseomonas mucosa* was decreased in rosacea patients compared to healthy controls [55].

## 4. Microbiome-Based Products and Skin Health—Treatment Approaches

**Figure 2:**
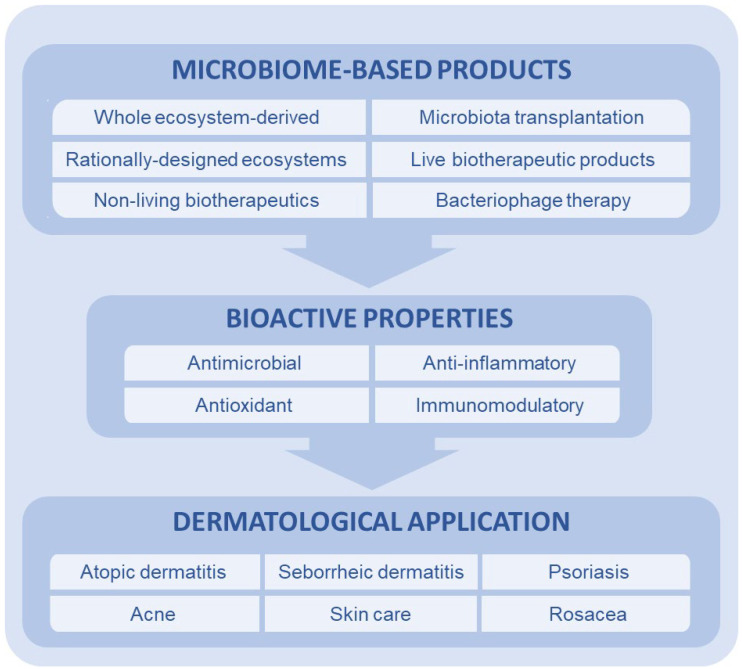
Overview of microbiome-based products (MBPs) and their bioactive properties and applications. MBPs range from less characterized, donor-dependent approaches like microbiota transplantation and whole ecosystem-based products, to highly defined and controlled interventions such as rationally designed microbial consortia, live biotherapeutic products (LBPs), non-living biotherapeutics, and phage therapies.

There are many definitions and classifications of products containing microorganisms or their components, which can provide health benefits or be used as therapeutic agents [110]. Initially, the World Health Organization (WHO) defined probiotics as “live microorganisms that, when administered in adequate amounts, confer a health benefit on the host” [12]. However, this definition covers a wide range of applications, from food products and dietary supplements to medicines. To provide a more precise definition for products with medical applications, the Food and Drug Administration introduced the category “Live Biotherapeutic Products” (LBPs) [111]. This category specifically refers to the use of live microorganisms as therapeutics, but excludes inanimate microbes, their metabolites and components (collectively referred to as postbiotics) and bacteriophages. To comprehensively include all the products that are based on and derived from microorganisms with the potential to provide health benefits (Figure 2) and in light of the fact that the terminology is constantly evolving, the term “microbiome-based products (MBPs)” was used throughout this review.

As the general knowledge about the role of the microbiome in various skin conditions continues to expand, modulating the immune system by restoring microbial homeostasis has emerged as a promising area of research. One direct approach to achieving this balance involves the use of the MBPs, both orally and topically [2,112]. Over the past decade, there has been a significant increase in the use of MBPs in skincare products and dermatological therapies [2]. These treatments have shown potential in the prevention and management of inflammatory skin diseases, potentially offering an alternative to traditional topical or systemic steroids and antimicrobial agents. However, clinical trials and efficacy studies in this field are still relatively limited. Although the exact mechanisms by which MBPs improve skin health are not yet fully understood, numerous studies have demonstrated their beneficial effects [80,113,114,115].

### 4.1. Topical MBPs

Standard therapeutic approaches for inflammatory skin diseases typically involve the application of corticosteroids (AD and psoriasis) [113,116] and antibiotics (acne) [4,16]. While these treatments are effective in managing the disease and alleviating symptoms, their mechanisms of action and associated side effects limit their long-term use. As a result, they provide short-term symptom relief rather than offering a long-term solution.

In recent years, there has been a growing interest in the application of natural microbiota-based formulations as topical treatments to re-establish microbial homeostasis, restore the skin barrier function and maintain the physiological balance between the host and its microbiota [113]. These formulations are generally considered safe, with fewer adverse effects compared to standard therapies for skin diseases [117]. Topical MBPs may include live or inanimate microorganisms, fermented lysates and extracts, all designed to modulate the skin microbiota and promote skin health. Initially, most research focused on the impact of gut-targeted MBPs on skin conditions [56,118], while more recent studies have shifted the therapeutic strategy toward the direct topical application of MBPs [2,113,115,117,119,120]. Several topically applied MBPs have shown positive effects against inflammatory skin diseases, such as improving the skin barrier, producing antimicrobial peptides, and reducing inflammation. Lactic acid bacteria (LAB) are currently the most commonly used microorganisms in microbiome-based therapies due to their well-established safety profile and GRAS status. However, emerging research is increasingly exploring other bacterial species and bacteriophages as promising therapeutic options for inflammatory skin diseases.

The application of MBPs has been most extensively studied in the treatment of atopic dermatitis, with more limited research available on their use in seborrheic dermatitis. Di Marzio et al. found that the topical application of a cream containing sonicated *Streptococcus thermophilus* improved ceremide concentrations in the skin and resulted in the improvement of the symptoms in AD patients [121]. Two studies demonstrated that emollients containing *Lactobacillus sakei* extract [122] or heat-treated *Lactobacillus johnsonii* NCC 533 [123] inhibited the growth of *S. aureus*, also restoring the skin barrier and reducing symptoms in subjects with AD. A recent clinical study proved that a topical ointment containing live *Lactobacillus reuteri* DSM 17938 decreased disease symptoms in AD patients after four and eight weeks of continuous use, showing great potential as a novel topical product [124]. In addition to LAB, other bacterial species have also been studied for their beneficial effects on human skin. Several reports describe positive effects of *Roseomonas mucosa* (commensal skin bacteria) on alleviating AD symptoms. Myles et al. showed that topical treatment containing live *R. mucosa* was associated with a significant reduction in AD severity, topical steroid requirement and *S. aureus* colonization on the skin in children with AD [125]. Another study developed a living bacterial formulation that combines live *R. mucosa* with a polymeric matrix into a skin product, which demonstrated several positive effects on the skin, including a reduction in *S. aureus* proliferation and the alleviation of immune/inflammatory responses associated with AD in an animal model [126]. Silverberg et al. conducted a clinical study which assessed safety and efficacy of a topical spray including live ammonia-oxidizing bacterium *Nitrosomonas eutropha*, reporting reduced pruritus and improved eczema symptoms in patients with mild to moderate AD [127]. Several interesting clinical studies showed that topically applied extract and lysate of *Vitreoscilla filiformis* (bacterium isolated from sodium sulfurized thermal waters) significantly reduced the severity of AD and SD [128,129,130]. Another clinical trial investigated the efficacy of a topical treatment containing *Lactobacillus* extract in reducing dandruff, a condition often associated with SD. After two weeks of application, the treatment was shown to improve overall scalp health [131]. In a recent clinical study, Truglio et al. investigated the effects of a topical oily suspension containing *Lactobacillus crispatus* P17631 and *Lacticaseibacillus paracasei* I1688 (EUTOPLAC formulation) on the skin microbiome and its potential to reduce SD severity, reporting an improvement in symptoms, with significant reductions in the *Malassezia* genus in patients with SD [132].

Phage therapy shows promising, though still preliminary, effectiveness in treating atopic dermatitis, with encouraging results primarily from small-scale studies and animal models. Experimental evidence for its potential in treating AD was obtained in the sensitized and *S. aureus* challenged mouse skin model where *S. epidermidis* was topically administered with or without *S. aureus* phage SAGU1. The groups treated with the phage showed the greatest reductions in *S. aureus* CFUs and plasma IgE levels [133]. Similarly, but more thoroughly, Geng et al. showed that the topical application of the novel *S. aureus* phage SAP71 improved skin lesions, reduced bacterial loads in the skin, and prevented the development of AD-like skin pathological changes in an AD model [134].

In addition to AD and SD, several studies have also explored the use of topical MBPs in acne treatment. Lebeer et al. developed a topical product incorporating live lactobacilli (*Lacticaseibacillus rhamnosus* GG, *Lactobacillus plantarum* WCFS1, and *Lactiplantibacillus pentosus* KCA1) and demonstrated a positive impact on acne symptoms in a clinical trial involving patients with mild-to-moderate acne. This formulation showed potential in modulating the skin microbiota, including a reduction in the relative abundance of staphylococci and *C. acnes* [135]. In a recent study, Podrini et al. investigated the effects of topically applied serum containing live *Lactobacillus plantarum* in a 2D human primary sebocyte culture as a model for treatment of mild-to-moderate papulopustular acne. The study demonstrated a significant reduction in the production of inflammatory mediators and a decrease in the abundance of acne-associated pathogens, including the class-A *C. acnes* [136]. Additionally, two clinical studies have demonstrated the effectiveness of *L. plantarum* lysates in treating mild-to-moderate acne and reducing acne lesions [137,138]. Majeed et al. report a clinical study demonstrating that a cream containing cell-free supernatants (CFS) of *Bacillus coagulans* and inactivated cells of *Bacillus longum* was effective in treating acne lesions and other seborrheic conditions [139]. Interestingly, another study demonstrated that using a lotion with CFS of *Enterococcus faecalis* SL-5 (lactic acid bacteria isolated from human feces) significantly reduced acne compared to placebo. This may be due to enterocins, antibacterial molecules produced by *E. faecalis* [140].

Bacteriophage therapy to treat acne vulgaris has emerged as a potential therapeutic solution. In murine models with *C. acnes*, bacteriophage injections reduced inflammation, epidermal thickness, and microcomedone-like cysts [141], while topical phage treatment decreased inflammatory and apoptotic markers and was effective against multidrug-resistant *C. acnes* [142,143]. An MBP containing a three-phage cocktail topical gel formulation was not irritant to human skin or ocular tissues in the ex vivo models and did not permeate through human epidermis while in a cosmetic clinical study, phage gel was well tolerated and reduced the facial burden of *C. acnes* [144].

On the other hand, the application of topical MBPs for the treatment of psoriasis and rosacea still remains underexplored. To our knowledge, there is only one animal study that has confirmed the effect of probiotic *Lactobacillus sakei* proBio-65 extract on the severity of skin inflammation in a mouse model of psoriasis [145]. There have been some reports suggesting that bacteriophage therapy could hold promise for treating psoriasis by addressing cutaneous bacterial dysbiosis and modulating immune responses [50,146,147] but firm experimental evidence is still missing. Despite the growing market for skincare products, many of which claim effectiveness in managing these conditions, there is a significant lack of scientific research and clinical trials to validate the efficacy and safety of these topical treatments.

Due to their growing popularity, a number of skincare products based on the microbiota can be found on the market. These products may contain live bacteria, inactivated bacterial cells, or their components (e.g., extracts, lysates and cell-free supernatants). Examples of products available on the market, as well as their effect on skin, are listed in Table 3. The listed products were selected based on the availability of reliable sources, including official websites, that clearly disclosed the formulation composition, the specific microbial strains used, and the claimed dermatological effects.

### 4.2. Oral MBPs

The gut microbiota plays a crucial role in regulating the host’s immune system, and gut microbiota dysbiosis can trigger autoimmune and inflammatory conditions, not only within the intestines but also in other organs, including the skin [2,118,150]. Growing evidence indicates that gut microbiota dysbiosis is frequently associated with skin diseases such as atopic dermatitis, psoriasis, rosacea, and acne vulgaris, highlighting the potential of oral MBPs as a promising therapeutic strategy for managing these dermatological conditions [2,58,150]. Recent reports have highlighted the potential benefits of oral MBPs for skin health, with most studies focusing on treating atopic dermatitis and acne.

There is a number of animal studies using MBPs to improve AD symptoms in mouse models. Fecal microbiota transplantation modulated the gut microbiota homeostasis and affected the recovery from AD-related inflammation by affecting epidermal layer thicknesses and suppressing inflammatory cytokines [151]. Kim et al. investigated the beneficial effects of a combination therapy using *Bifidobacterium longum* and the prebiotic galactooligosaccharide (GOS), showing improvement in skin inflammation markers, TEWL and the deficiency of epidermal barrier proteins in the dinitrochlorobenzene (DNCB)-induced AD model [152]. Similar findings were reported for the oral administration of *L. paracasei* subsp. *paracasei* NTU 101 [153], tyndallizated *L. rhamnosus* IDCC 3201 [154], and *Lactobacillus sakei* WIKIM30 isolated from kimchi [155]. One study proved that the ingestion of yoghurt containing *Lactococcus lactis* 11/19-B1 significantly improved the severity of AD symptoms in a mouse model and in a clinical trial conducted on children suffering from AD [156]. There are several clinical trials showing that the combination of different lactic acid bacteria species with or without a prebiotic component can reduce the severity of AD symptoms. For instance, Navarro-López et al. investigated the effect of the oral intake of a capsule containing *Bifidobacterium lactis* CECT 8145, *Bifidobacterium longum* CECT 7347, *L. casei* CECT 9104 and maltodextrin (prebiotic) and reported efficacy in reducing the SCORAD index and reducing the use of topical steroids in children with moderate AD [157]. Another clinical study conducted on children with moderate-to-severe AD showed significant clinical improvement in AD severity after therapy containing a probiotic mixture of *L. paracasei* and *Lactobacillus fermentum* [158]. Although most studies are conducted on lactic acid bacteria due to their proven safety (GRAS status), recent research aimed to prove the positive effect of other species of human microbiota. Lee et al. used animal models to assess the beneficial effect of commensal gut bacteria *Faecalibacterium prausnitzii* and *Akkermansia muciniphila* in the pathogenesis of AD. The study resulted in improvements in AD-related markers and a reduction in TSLP levels, which suggest that these strains have therapeutic potential in AD [159].

In addition to AD, the use of oral MBPs has been most explored for acne treatment. Several in vitro studies have demonstrated the antibacterial activity and anti-inflammatory effects of oral MBPs, which may be beneficial in the treatment of acne [160,161,162,163,164]. Furthermore, Espinoza-Monje et al. proved the antimicrobial and immunomodulatory properties of lactic acid bacterium *Weissella viridescens* UCO-SMC3 and demonstrated its beneficial effect in the treatment of acne vulgaris in vitro and in a mouse model [165]. As for clinical trials, a recent study from Eguren et al. reported the oral application of a capsule containing *L. rhamnosus* (CECT30031) and the cyanobacterium *Arthrospira platensis* (BEA_IDA_0074B) as a safe and effective with clinical and statistically significant reduction in the severity of acne compared to placebo [166]. Another study tested the efficacy of a dietary supplement containing a mixture of *Bifidobacterium breve* BR03 DSM 16604, *L. casei* LC03 DSM 27537, *Ligilactobacillus salivarius* LS03 DSM 22776, and a botanical extract (containing lupeol from *Solanum melongena* L. and *Echinacea* extract) in patients with mild to moderate acne. The results from this study provide a promising alternative for the treatment of inflammatory acne as well as for the control of acne-prone skin [167]. In addition to the studies mentioned above, there are several clinical trials that have applied a combination of therapies including MBPs and standard treatments (e.g., antibiotics). Jung et al. conducted a study in acne patients using a probiotic mixture containing *L. acidophilus, L. bulgaricus and B. bifidum*, applied with or without minocycline. After 12 weeks of treatment, patients treated with the probiotic mixture plus minocycline showed significant improvement in their acne lesions [168]. Interestingly, another clinical study involving patients with intestinal-related dermatoses, including acne, explored the effects of strain *E. coli* Nissle 1917, and found that the patient group receiving the bacterium orally along with conventional topical therapy (tetracycline, steroids, and retinoids), showed significant improvement compared to patients in the conventional therapy-only group [169].

As previously noted, there is a lack of human studies examining the use of orally applied MBPs for other inflammatory skin conditions, such as SD, psoriasis, and rosacea. Chen et al. investigated the effect of orally administered *Lactobacillus pentosus* GMNL-77 in a psoriasis-like skin inflammation animal model and demonstrated that the therapy significantly reduced erythematous scaling lesions, a symptom characteristic for psoriasis [170]. A clinical study demonstrated the efficacy of orally administered *L. paracasei* NCC 2461 ST11 in managing dandruff (symptom typical for SD) and restoring a balanced scalp microbiota [171]. Research into the use of oral MBPs for the treatment of rosacea is increasingly gaining attention in clinical practice. For instance, Fortuna et al. conducted a case study on a rosacea patient who underwent a treatment of low-dose doxycycline alongside probiotic therapy, which included *Bifidobacterium breve* BR03 and *L. salivarius* LS01. Following the treatment, the patient displayed significant improvement in both cutaneous and ocular manifestations, leading the researchers to recommend discontinuing doxycycline and continuing with probiotic therapy [172].

### 4.3. Regulatory Landscape

The regulatory framework for microbiome-based products is developing quickly, driven by growing scientific insights and commercial interest. The regulatory classification depends heavily on the product’s intended use, whether for therapeutic, nutritional, or cosmetic purposes, which directly affects how it is reviewed and approved [15,173,174]. Although the rapid pace of scientific discoveries is well reflected in microbiome-based innovations and product development, regulatory alignment has yet to fully keep pace and account for the complexity and novelty of these products.

Consumer microbiome products, such as probiotics, dietary supplements, and personal care items, are usually regulated under food or cosmetic laws. These categories typically face limited pre-market oversight, leading to concerns about product quality, labelling accuracy, and consumer benefits and safety. In case therapeutic claims are made, they may be reclassified as drugs.

Microbiome-based therapies, particularly live biotherapeutic products (LBPs), are emerging as novel medical treatments designed to modulate the microbiome, improve health or treat disease. These products are generally regulated as drug or medicinal products by agencies such as the U.S. Food and Drug Administration (FDA) and the European Medicines Agency (EMA). Regulatory requirements for LBPs and similar therapies include extensive documentation on identity, purity, potency, and safety, as well as robust preclinical and clinical trial data. Microbiome-based therapies vary in complexity and donor dependence ranging from fully complex like fecal microbiota transplantation, whole microbial ecosystems, and rationally designed microbial consortia, to simpler ones like LBPs (single-strain or mixtures of multiple strains), non-living biotherapeutic products and bacteriophages [15]. The legal definition and regulatory pathway for authorizing MBPs remain key challenges for scientists, regulatory authorities, and product developers. For complex products, the origin of the microbial material is critical in determining the benefit–risk ratio, whereas for less complex products, thorough characterization and quality control play a more significant role.

As knowledge is increasing and new products advance, regulatory frameworks need to evolve to reflect the challenges of product design and manufacturing, delivery methods, and targeted indications. The U.S. has taken the lead with the approval of therapies for recurrent *Clostridium difficile* infection, while in the European Union there is a lack of harmonization regarding the regulatory frameworks applied to various MBPs but progress is expected through updated frameworks such as the proposed Substances of Human Origin (SoHO) legislation [15]. However, no topical microbiome therapeutic products for treating skin disease have been approved to date [113,117] and most topical formulations are still primarily used as personal skincare products [104,108].

Still, numerous scientific and regulatory hurdles remain, such as defining what constitutes a “healthy” microbiome, accounting for inter-individual variability, understanding complex host–microbiome interactions, evaluating long-term safety and validating/standardizing analytical methodology, strain isolation and banking as well as controlling manufacturing processes. Addressing these challenges will require continued collaboration among regulators, scientists, and industry stakeholders to ensure that safe, effective microbiome-based therapies reach patients and consumers worldwide.

## 5. Conclusions

MBPs represent a promising therapeutic approach for inflammatory skin diseases such as atopic dermatitis, seborrheic dermatitis, acne, psoriasis and rosacea. These formulations aim to restore microbial homeostasis and modulate immune responses, addressing the underlying pathophysiology of these chronic diseases. The advantages of MBPs are compelling, as they offer a multifaceted mechanism of action, including the modulation of the gut–skin axis, the enhancement of skin barrier function, and reduction in systemic inflammation. Several clinical studies have demonstrated that MBPs can reduce disease severity and inflammatory markers. In addition, MBPs generally exhibit favourable safety profiles, with minimal systemic absorption and a low risk of adverse effects.

However, several challenges must be addressed. The complexity of the skin microbiome and individual variability in microbiota composition complicate the development of standardized treatments. Furthermore, the long-term effects of microbiome modulation are not yet fully understood. Further research is needed to determine the mechanisms of action of MBPs, along with clinical trials to assess the safety and efficacy of topical formulations. Such advancements may pave the way for the development of the first topical MBPs as therapeutics for the treatment of inflammatory skin diseases.

Looking forward, the integration of personalized medicine, including microbiome profiling, holds promise for optimizing microbiome-based therapies. Additionally, interdisciplinary collaboration among dermatologists, microbiologists, and bioengineers will be essential to translate this promising science into clinically viable solutions. Further research is crucial to overcome existing limitations and fully harness the therapeutic potential of MBPs in managing inflammatory skin diseases.

## Figures and Tables

**Table 1:** Key terms used in this paper.

Term	Definition	Ref.
Microbiota	A community of microorganisms (bacteria, fungi, archaea and viruses) living within a specific environment (e.g., gut, oral, respiratory, and skin microbiota).	[9]
Microbiome	The collection of microorganisms, their genomes, structural elements and metabolites, and the specific environment in which they live.	[9]
Mycobiome	Total community of fungi and their genetic material present in a particular environment or host	[10]
Phageome	Total community of bacteriophages and their genetic material present in a particular environment or host	[11]
Probiotic	Live microorganisms that, when administered in adequate amounts, confer a health benefit on the host.	[12]
Prebiotic	Non-digestible food ingredients which selectively stimulate the growth and/or activity of microorganisms with a positive influence on the health of the host.	[13]
Postbiotic	Bioactive compounds produced by microbiota, their components and/or inanimate microorganisms which confer a health benefit to the host.	[14]
Microbiome-based product (MBP)	A wide range of products, from food to medicinal products, including food supplements, foods for special medical purposes, cosmetics or medical devices, based on complex living systems.	[15]

**Table 2:** Characteristics of human skin microbiota in inflammatory skin diseases.

Disease	Skin Microbiota Alternations		Ref.
	Increase	Decrease	
Atopic dermatitis	*Staphylococcus* spp. *Staphylococcus aureus* *Malassezia restricta*/ *Malassezia globosa* ratio *Staphilococcus aureus* phages	*Cutibacterium* spp. *Streptococcus* spp. *Acinetobacter* spp. *Corynebacterium* spp. *Prevotella* spp.	[30,36,37,38,39]
Seborrheic dermatitis	*Staphylococcus* spp. *Staphylococcus aureus* *Pseudomonas* spp. *Micrococcus* spp. *Malassezia* spp. *Acinetobacter* spp. *Streptococcus* spp.	*Cutibacterium* spp. *Corynebacterium* spp.	[40,41]
Acne	*Staphylococcus* spp. *Cutibacterium acnes* IA1	*Cutibacterium acnes* phages	[42,43,44,45]
Psoriasis	*Staphylococcus aureus* *Streptococcus pyogenes* *Corynebacterium kroppenstedtii* *Corynebacterium simulans* *Finegoldia* spp. *Malassezia* spp.	*Cutibacterium* spp. *Lactobacillus* spp. Bacteriophages	[46,47,48,49,50]
Rosacea	*Demodex folliculorum* *Bacillus oleronius* *Bacillus simplex* *Staphylococcus epidermidis* *Corynebacterium kroppenstedtii*	*Roseomonas mucosa*	[51,52,53,54,55]

**Table 3:** Selected commercial microbiota-based products for topical skin applications that contain viable microbial cells and/or their compounds (e.g., extract, ferment lysate, and supernatants).

No.	Product/Brand	Microbial Component	Benefits Claimed
Live bacteria			
1	Serum BLIS Q24 (Blis Technologies, New Zealand)	*Micrococcus luteus* Q24—strain isolated from skin of a healthy human adult	Balances the skin microbiome; reduces oiliness, acne, redness/rosacea, rough skin, wrinkles [148,149].
2	BAK Serum for acne-prone skin (BAK Skincare, Denmark)	*Lactobacillus plantarum* LB356R and LB244R—isolated from fermented cabbage and beets	Balances the skin microbiome; restores skin barrier; reduces the formation of *C. acnes* biofilm.
3	SkinDuo Probiotic Topical Serum (The BioArte, Malta)	*Lactobacillus plantarum*	Balances the dysbiosis in hair follicles environment; reduces sebum production and inflammation [136].
4	Esse Probiotic Serum (Esse Skincare, South Africa)	*Lactobacillus* spp. isolated from human gut	Complements natural microbial diversity; strengthens the skin barrier.
5	Squalane + Probiotic Gel Moisturizer (Biossance, USA)	*Lactobacillus, Lactococcus, Leuconostoc, Pediococcus, Saccharomyces, Hansenula*	Supports a healthy microbiome for balanced skin. Suitable for oily, acne-prone, or sensitive skin.
6	Youthful Serum (Gallinée, UK)	*Lactobacillus crispatus* + *Lactobacillus* ferment lysate	Improves skin hydration and balances skin microbiome.
Inanimate bacteria/extract/ferment lysate/cell-free supernatants			
1	LactoSporin (Sabinsa Cosmetics, USA)	Cell-free supernatant of *Bacillus coagulans* and inactivated cells of *Bacillus longum*	Effective against mild-to-moderate acne and other seborrheic conditions [139].
2	Probiotic Cream (NiKEL, Croatia)	*Lactobacillus* ferment	Regenerates damaged skin, reduces inflammation, and accelerates skin recovery. It is suitable for conditions like redness, flaking, and seborrheic dermatitis, and can complement psoriasis treatments.
3	The Probiotic Concentrate (Aurelia, UK)	*Bifida* ferment	Strengthens skin immune system and protect the skin barrier. Reduces skin irritation and inflammation. Balances skin microbiome.
4	Probiotic Concentrate (Columbia skincare, USA)	*Lactococcus* ferment lysate	Enhances the skin’s renewal process.
5	Lipikar Eczema Cream (La Roche Posay, France)	*Vitreoscilla* ferment	Visibly reduces signs of eczema and provides long-lasting relief. Helps relieve itching and irritation.
6	Advanced Génifique serum (Lancome, Australia)	*Bifida* ferment lysate	Strengthens skin barrier function. Balances skin microbiome.

## Abbreviations

The following abbreviations are used in this manuscript:

AD	Atopic dermatitis
AMP	Antimicrobial peptides
CFU	Colony forming units
FDA	Food and Drug Administration
GRAS	Generally Recognized as Safe
ISDs	Inflammatory skin diseases
LBP	Live biotherapeutic product
MBP	Microbiome-based product
SCORAD	Scoring atopic dermatitis
SD	Seborrheic dermatitis
